# CO-DESIGNING A GREEN ACTIVITY PRESCRIPTION PROGRAM WITH HISPANIC/LATINO PERSONS LIVING WITH MEMORY CHALLENGES

**DOI:** 10.1093/geroni/igad104.3230

**Published:** 2023-12-21

**Authors:** Rebecca Lassell, Valeria Tamayo, Triana Pena, Carlos Tejeda, Anderson Torres, Jessica Zwerling, Laura Gitlin, Abraham Brody

**Affiliations:** Indiana University School of Public Health-Bloomington, Bloomington, Indiana, United States; New York University, New York City, New York, United States; New York University, New York City, New York, United States; Montefiore, Bronx, New York, United States; R.A.I.N. Total Care, Inc., Bronx, New York, United States; Montefiore, Bronx, New York, United States; Drexel University, Philadelphia, Pennsylvania, United States; New York University, New York City, New York, United States

## Abstract

Nature activities can improve well-being and may modify risk factors for cognitive decline. There is a gap between nature activities offered within a person’s community and their ability to participate in them, including Hispanic/Latino persons living with memory challenges (MCI and mild dementia) in urban lower-resourced areas. An occupational therapist can bridge this gap by tailoring nature activities available in a person’s home, neighborhood, and community to their interests and needs. Utilizing a participatory approach, we sought to co-design a 12-week Green Activity Prescription (GAP-H) with Hispanic/Latino individuals living with memory challenges and their care partners. Participants were recruited via convenience and snowball sampling in the Bronx, NY with outdoor activity professionals (n=7), Hispanic/Latino persons living with memory challenges and care partners (n=5), and interdisciplinary healthcare providers/dementia experts (n=7). Co-design occurred iteratively with 5 focus groups and 2 individual interviews lasting 30-90 minutes and focused on intervention and research design. Sessions were recorded and transcribed. Utilizing directed content analysis data was coded using a priori codes intervention design and research design. Participant preferences for intervention design were captured by subcodes session duration (30-90 minutes), frequency (4-8 sessions), and delivery modes (in-person and phone). Participants’ preferred nature activities included group exercise and outdoor crafts [crocheting], outcomes of well-being, social participation, decreased loneliness, and stewardship were identified. Preferred language for recruitment materials were memory challenges, Hispanic/Latino, and well-being. Referral pathways were identified including community-based organizations and primary care. Findings can inform the co-design process of other interventions for this population.

